# Synchronous Acute Cholecystitis and Perforated Acute Appendicitis in a 49-Year-Old Male Patient in Jordan: A Case Report

**DOI:** 10.7759/cureus.114269

**Published:** 2026-08-10

**Authors:** Rayyan Al-Qaryouti, Murad M Hamiedah, Omar Makhamreh, Rawnaq Al-Qaryouti, Ahmad Shurbaji, Yousef Banat, AlMiqdad Al-Faouri

**Affiliations:** 1 General Surgery, Jordanian Royal Medical Services, Amman, JOR; 2 Plastic Surgery, Jordanian Royal Medical Services, Amman, JOR; 3 Medicine, University of Jordan, Amman, JOR

**Keywords:** abdominal imaging, acute appendicitis, acute cholecystitis, case report, jordan, perforated appendix, surgical site infection, synchronous presentation

## Abstract

Acute appendicitis (AAP) and acute cholecystitis (AC) are two of the most common surgical emergencies, yet their synchronous occurrence is exceptionally rare and diagnostically challenging. We report the case of a 49-year-old male patient who presented with a 10-day history of diffuse abdominal pain, nausea, vomiting, anorexia, and dark urine. Examination revealed abdominal tenderness with positive Murphy's and rebound signs. Laboratory tests demonstrated leukocytosis, neutrophilia, and elevated liver enzymes. Imaging with combined ultrasound and computed tomography confirmed both an inflamed, stone-bearing gallbladder and an enlarged, fluid-filled appendix. Diagnostic laparoscopy revealed active pelvic purulence and a severely inflamed gallbladder with dense inflammation at the hepatocystic triangle. Prioritizing definitive source control while avoiding iatrogenic biliary manipulation in a contaminated field, cholecystectomy was deferred. The patient underwent an appendectomy, which was converted to an open gridiron approach due to severe adhesions and tissue friability obscuring the appendiceal base. Index histopathology confirmed perforated AAP with serositis. Postoperatively, the patient was readmitted on day 14 with a superficial incisional surgical-site infection; this was successfully managed with bedside drainage and antibiotics after imaging ruled out deep intra-abdominal collections. Three months later, the patient underwent an uncomplicated interval laparoscopic cholecystectomy, with final histopathology confirming chronic cholecystitis. The rarity of this presentation highlights the need for high clinical suspicion. Synchronous perforated AAP with AC remains a rare diagnostic challenge in atypical abdominal pain. Early multimodal imaging and individualized operative planning, including staged intervention when severe inflammation or unsafe anatomy precludes simultaneous resection, may be considered to optimize patient safety.

## Introduction

Acute appendicitis (AAP) and acute cholecystitis (AC) are among the most frequent causes of acute abdomen worldwide. Each condition has a well-established clinical presentation and diagnostic pathway, yet their synchronous occurrence remains exceptionally rare. Pathophysiologically, this rarity stems from their distinct etiologies and anatomical origins; appendicitis typically results from appendiceal luminal obstruction (e.g., by a fecalith or lymphoid hyperplasia), whereas AC usually arises from gallstone impaction in the cystic duct. Because these inflammatory processes operate via independent mechanisms in different abdominal compartments, their simultaneous presentation is highly unusual. Typical appendicitis presents with migrating periumbilical pain that localizes to the right lower quadrant, fever, leukocytosis, and gastrointestinal upset, whereas AC more commonly manifests with right upper quadrant pain, positive Murphy’s sign, fever, and abnormal inflammatory or liver function tests [1].

Despite the commonality of each disease individually, their simultaneous presentation is diagnostically challenging and often recognized only after detailed imaging or intraoperative findings [2,3]. The clinical importance of recognizing this dual pathology cannot be overstated. A missed secondary diagnosis risks persistent postoperative sepsis and emergent reoperation, while early preoperative identification fundamentally alters operative planning, dictating optimal trocar placement, surgical approach, and the decision between simultaneous resection versus a staged intervention. Cross-sectional imaging, particularly ultrasound in combination with computed tomography, plays a decisive role in identifying dual pathology [4]. Recent literature reviews and case reports have emphasized that laparoscopic cholecystectomy with appendectomy is feasible in most patients; however, open or staged approaches remain necessary in selected cases where inflammation or adhesions limit operative safety [5,6].

In this case report, we describe the case of a 49-year-old male who presented to our emergency department with diffuse abdominal pain later localizing to both the right upper and lower quadrants, associated with systemic and gastrointestinal symptoms. Imaging confirmed synchronous AAP and AC. Intraoperatively, we found a perforated appendix with surrounding pus and a severely inflamed, stone-bearing gallbladder with extensive adhesions, leading us to perform an appendectomy and defer cholecystectomy. We compare this case with the available literature, highlighting similarities and distinctive features in terms of demographics, clinical presentation, diagnostics, operative management, and postoperative outcomes.

## Case presentation

Patient’s history

This is a case of a 49-year-old male patient with a medical history of newly diagnosed hypertension, currently on enalapril 20 mg Q-day, with no previous surgical history and a previous history of an allergic reaction to dexamethasone injection, who presented to the ER complaining of abdominal pain for 10 days. The pain initially started as vague and diffuse, but over the 10 days, it evolved into two distinct focal areas of maximal intensity in the right lower and right upper quadrants. It was sharp and stabbing in nature, radiating to his back and left shoulder - a classic referred pain to the left shoulder, indicating diaphragmatic irritation likely secondary to extensive peritoneal inflammation. The patient visited outpatient clinics three times during the timeframe of his symptoms. During these visits, he was managed symptomatically with intravenous (IV) fluids and analgesics for suspected gastroenteritis or simple biliary colic without undergoing cross-sectional imaging. This symptomatic treatment likely masked the progressive peritonitis of his dual pathology and contributed to the delayed definitive diagnosis.

Examination and ER management

On examination, the patient was conscious, alert, and oriented to time, place, and person. He was febrile, with a temperature of 38 °C documented in the ER using a sublingual thermometer. His heart rate was 86 bpm, and his blood pressure was 130/80 mmHg. His abdomen was soft and lax, with involuntary guarding, and two distinct areas of focal tenderness were noted, associated with a positive Murphy’s sign, positive rebound tenderness, positive Rovsing’s sign, and a negative obturator sign. His Alvarado score was highly indicative of AAP (score of 8: migrating pain, anorexia, nausea/vomiting, RLQ tenderness, rebound, fever, leukocytosis, and left shift). Concurrently, his presentation met the Tokyo Guidelines (TG18) criteria for Grade II (moderate) acute cholecystitis, given the duration of symptoms exceeding 72 hours and marked local inflammation, but without Grade III organ dysfunction.

Resuscitation with IV fluids, 500 mL of 0.9% normal saline, was performed in addition to hyoscine butylbromide and metoclopramide to control the nausea, vomiting, and pain. Complete blood count, kidney function tests, liver function tests, and urinalysis were obtained from the patient. The results showed leukocytosis with a neutrophilic shift, with a rise in his liver function tests (institutional reference ranges: total bilirubin ≤ 1.2 mg/dL, alkaline phosphatase (ALP) ≤ 129 U/L, aspartate aminotransferase (AST) ≤ 37 U/L, alanine aminotransferase (ALT) ≤ 41 U/L) (Table 1). Additional tests such as amylase, lipase, and gamma-glutamyl transferase (GGT) were not drawn in the emergency setting. Blood cultures and viral hepatitis screening were also omitted, as the patient’s clinical presentation and subsequent cross-sectional imaging provided a definitive and immediate intra-abdominal source for his systemic inflammatory response.

**Table 1:** Timeframe of patient laboratory testing. WBC, white blood cells; HB, hemoglobin; HCT, hematocrit; ALT, alanine transaminase; AST, aspartate transaminase; ER, emergency room; POD, postoperative day; NA, not available

Lab test	Outpatient clinic readings 5 days before ER visit	First ER visit/op day	POD 1	POD 2	POD 3	POD 4	POD 5	Discharge day	Reference range, unit
WBC	10.9	15	23.2	20.9	15.9	14.8	13.5	10.63	3.4-9.6 x 10^3/uL
Neutrophils %	97.2	69.5	90.1	78.6	80.5	80.6	76.7	71.9	40%-60%
Hb	16.9	14.8	14.4	13.1	12.5	12.5	12.2	12	13.2-16.6 g/dL
HCT	48.8	43.2	43.3	40.5	36.4	36.7	36.4	37.1	38.3%-48.6%
ALT	NA	124	89.8	141	93.9	72.6	72.8	64	<=41 U/L
AST	NA	86	62.9	125	70	51	58	57	<=37 U/L
Total bilirubin	NA	2.34	1.54	1.57	1.62	1.19	1.09	1.11	0.1-1.2 mg/dL
Direct bilirubin	NA	0.47	NA	0.18	0.67	0.24	0.24	0.41	0.01-0.3 mg/dL
Alkaline phosphatase	NA	497	377	533	420	350	315	249	40-129 U/L

Imaging

Abdominal ultrasound was performed in the ER (images not available for publication). The formal report indicated that his gallbladder was partially contracted, with a thick wall measuring 5 mm and containing a 1 cm stone; pericholecystic fluid was also noted. No intrahepatic or extrahepatic biliary dilatation was seen, and the common bile duct was normal in diameter. Pelvic free fluid was seen, but the appendix could not be visualized on ultrasound.

**Figure 1:**
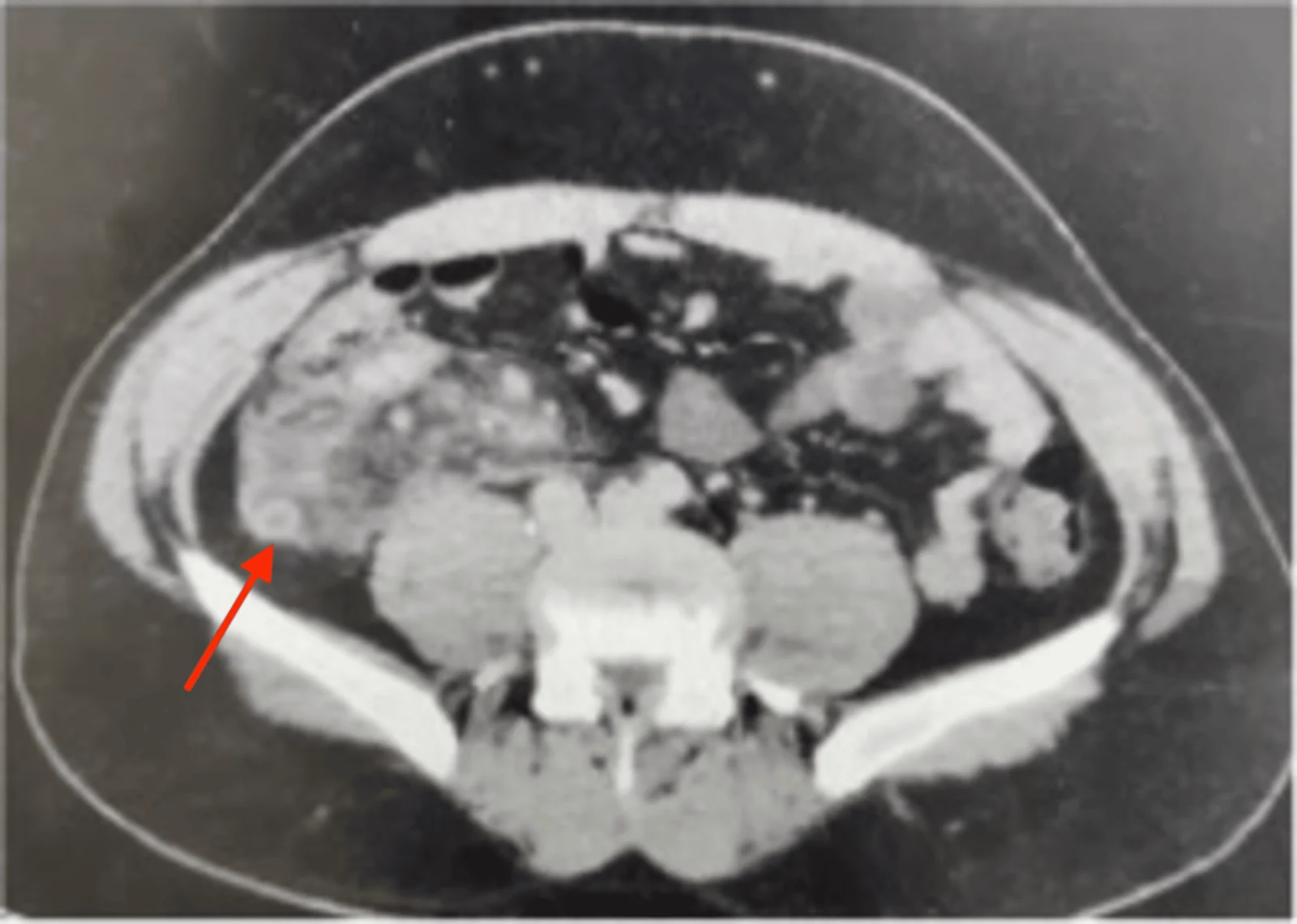
Appendix on CT scan, axial view. The red arrow indicates the inflamed appendix surrounded by significant fat stranding.

**Figure 2:**
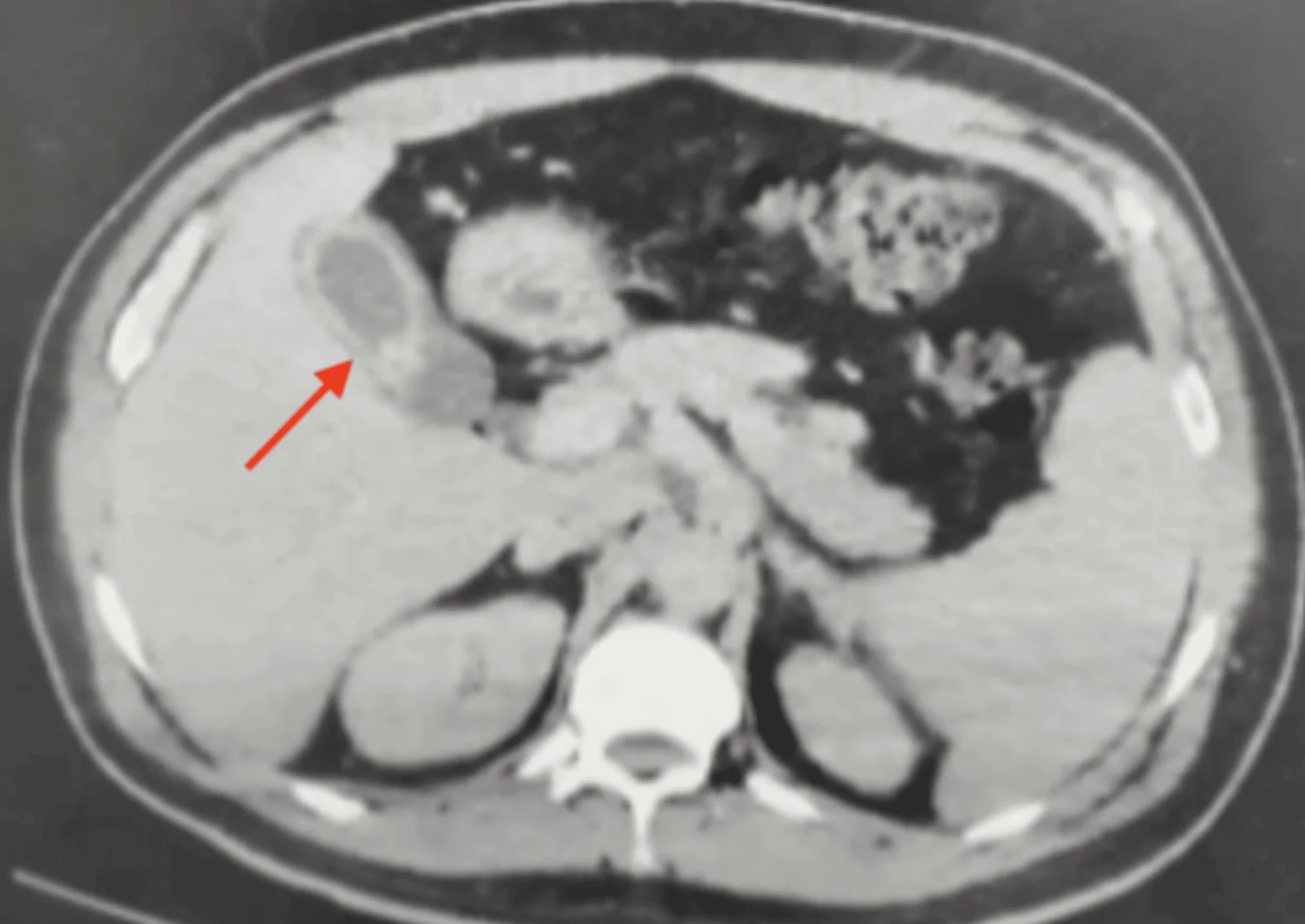
Gallbladder on CT scan, axial view. The red arrow indicates a thickened gallbladder wall with pericholecystic fluid and a stone.

**Figure 3:**
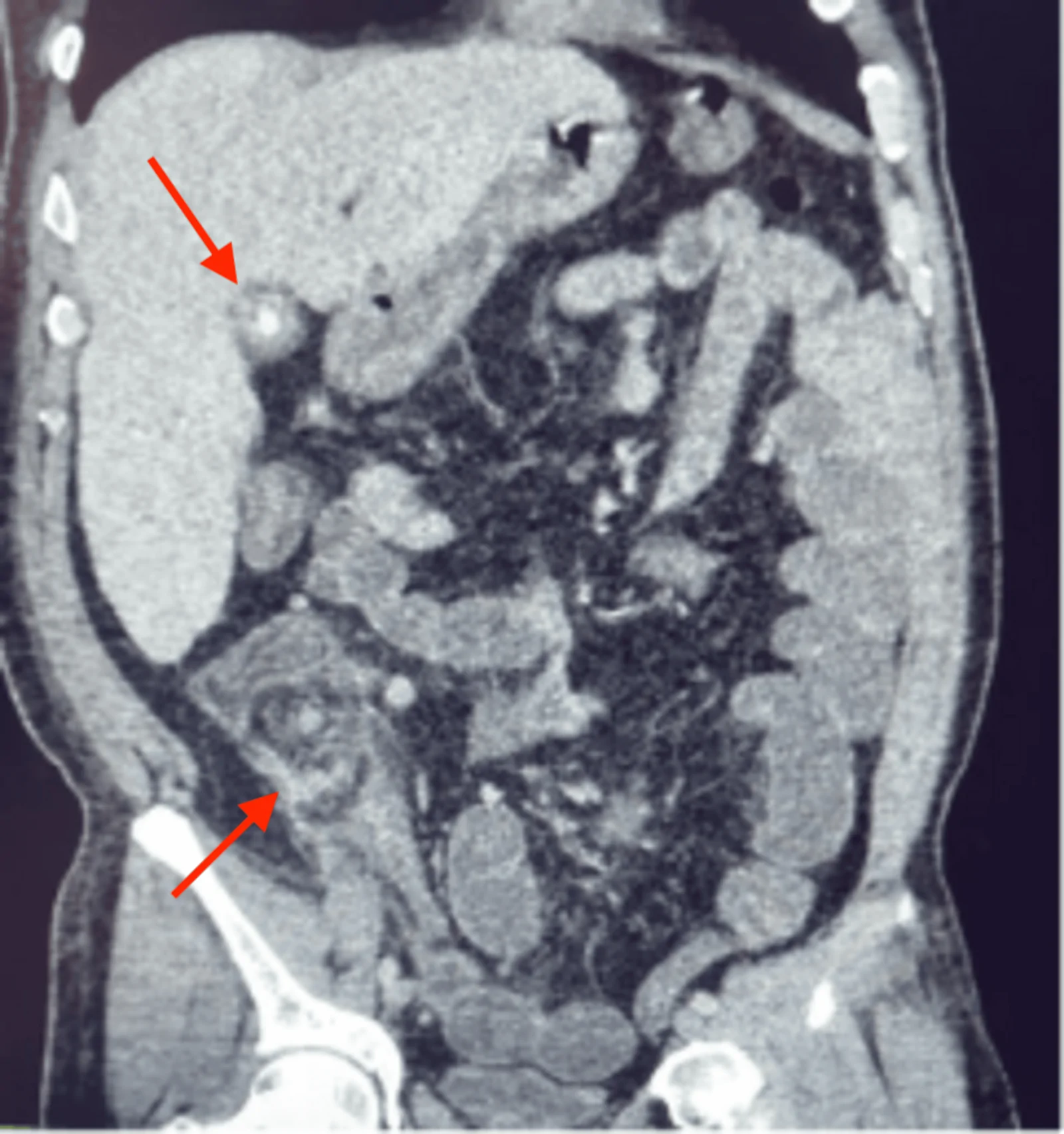
Synchronous view of the inflamed gallbladder and appendix on CT scan, modified coronal view. The upper arrow indicates the gallbladder with a stone and thickened wall. The lower arrow indicates the inflamed appendix.

An abdominal CT scan was performed, and the appendix appeared to be fluid-filled, measuring about 1 cm in AP diameter, surrounded by significant fat stranding, multiple lymph nodes, and free fluid. The gallbladder was surrounded by pericholecystic fluid, and a 1 cm stone was seen inside it, with no intrahepatic or extrahepatic biliary dilatation. Given the absence of biliary ductal dilatation on both ultrasound and CT, and because the patient's total bilirubin levels began trending downward promptly following initial resuscitation, further biliary imaging such as magnetic resonance cholangiopancreatography (MRCP) was not indicated (Figures 1-3).

Operation

A full diagnostic laparoscopy was initially performed to assess the abdomen and the extent of peritoneal contamination. Alongside the perforated appendicitis and pelvic purulence, the gallbladder was found to be severely inflamed and distended, with a thick edematous wall embedded in omental adhesions. However, there was no visual evidence of frank gangrene, macroscopic perforation, or gross empyema. Given the active peritoneal contamination, definitive source control via appendectomy was prioritized. Laparoscopic cholecystectomy was deferred to avoid manipulation of the biliary tree in a purulent field. Because the gallbladder remained intact without signs of necrosis, and the clinical picture did not meet the Tokyo Guidelines criteria for severe (Grade III) acute cholecystitis with organ dysfunction, we concluded that conservative management with IV antibiotics was appropriate.

Attention was then turned to the appendectomy. Despite initial laparoscopic mobilization, severe inflammatory adhesions densely plastered the cecum and obscured the appendiceal base. The surrounding tissues were highly friable, making safe laparoscopic dissection and secure closure of the appendiceal stump hazardous. Consequently, the procedure was converted to an open approach via a gridiron incision. The pelvic and right lower quadrant abscesses were evacuated, copious peritoneal irrigation was performed, and a sample was sent for microbiological culture. The perforated appendix was excised and sent for histopathology, and a surgical drain was placed in the pelvis. Postoperatively, the patient was managed with maintenance IV fluids, analgesics, and an antibiotic regimen of ceftazidime and metronidazole.

Follow-up

On day 1 postoperatively, the patient was seen in the surgical ward; his pelvic drain contained 100 cc of serosanguineous fluid. On day 2 postoperatively, although the drain output was 70 cc, the fluid remained strictly serosanguineous and nonpurulent, with no enteric or bilious contents. Because the patient was clinically improving, was afebrile, ambulating, tolerating oral intake, and had a reassuring abdominal examination, the decision was made to remove the drain. The dressing was clean, and the patient was well overall. Laboratory results were as shown in Table 1.

On day 3 postoperatively, the patient's average BP over the day was 160/90 while he was taking his BP medication. He also developed right arm swelling and redness; therefore, an internal medicine consultation was requested to assess his BP and rule out DVT. An arm Doppler ultrasound was performed to rule out DVT, with negative findings (images not available). A new echocardiogram was performed and was normal. His BP medications were adjusted.

Days 4 and 5 passed with no noticeable events; the patient wanted to stay for one more day to ensure full recovery.

On day 6 postoperatively, the patient was fit for discharge and was instructed to return to the clinic for follow-up in two weeks. He was also informed about the potential need for elective cholecystectomy in three to six months.

The formal histopathology report confirmed the diagnosis to be acute perforated appendicitis with serositis (microscopic images not available).

Postop complications

Eight days after hospital discharge, the patient presented to the ER again, complaining of abdominal pain under the open appendectomy incision, associated with a fever of 38 °C and active purulent discharge from the wound. No changes in bowel habits or urinary symptoms were reported. His heart rate was 111 bpm, and his blood pressure was 110/85 mmHg. Based on the Centers for Disease Control and Prevention (CDC) criteria, this was diagnosed as a superficial incisional surgical site infection (SSI). In the ER, the wound was partially opened to facilitate adequate drainage of the pus, and a wound swab was sent for microbiological culture. Abdominal ultrasound and CT scan were performed, successfully ruling out any deep organ-space intra-abdominal abscess or fluid collection.The patient was admitted for three days and managed with daily wound dressings and IV antibiotics. He responded well to this conservative management; the wound base became clean and granulating, and he was subsequently discharged for outpatient follow-up.

At three months postoperatively, the patient was readmitted for an elective interval laparoscopic cholecystectomy. Intraoperatively, the gallbladder wall remained noticeably thick and edematous; however, the severe acute inflammation that had previously frozen the hepatocystic triangle had completely subsided. This allowed for safe dissection and attainment of the critical view of safety without complication. The final histopathology report confirmed chronic cholecystitis, reflecting resolution of his initial acute episode and providing definitive pathological correlation for his dual presentation.

## Discussion

Our patient's presentation of fever, anorexia, nausea, and vomiting, with pain localizing to both the right upper and lower quadrants, falls within the spectrum reported in the literature. In the systematic review by Tuncer et al., of 37 patients with detailed symptomatology, fever was present in 16, vomiting in 19, nausea in 16, and anorexia in 5 [4]. This overlap demonstrates how clinical findings alone rarely suggest synchronous pathology. Imaging, therefore, was critical. The abdominal ultrasound demonstrated a thickened gallbladder wall with a 1 cm stone and pericholecystic fluid, while computed tomography revealed an inflamed appendix (10 mm, surrounded by fat stranding) and gallbladder inflammation. Such a dual-modality approach reflects the literature, in which 14 of 37 patients underwent both US and CT, and another 7 were diagnosed by CT alone [2,4].

Demographically, our patient was a 49-year-old male. In the review of 40 patients, the sex distribution was equal (20 male, 20 female), with a median age of 41 years; the male subgroup had a median age of 50 years [4]. Thus, our case aligns closely with the male age distribution. Laboratory findings further supported the diagnosis: our patient had marked leukocytosis (up to 23.2 × 10^9^/L), a neutrophilic shift, and abnormal liver function tests. In the pooled series, leukocytosis was observed in 25 of 33 patients with laboratory data available [4]. Elevations in bilirubin and alkaline phosphatase, as we observed, have also been reported in synchronous presentations [2].

Our case highlights several critical diagnostic pitfalls and teaching points for synchronous presentations. First, the patient’s referred shoulder pain was a pivotal clue pointing to widespread diaphragmatic irritation from his ruptured appendix and pelvic free fluid, a finding that should elevate suspicion beyond localized disease. Second, the patient's three prior outpatient visits underscore how isolated symptomatic treatment without definitive imaging can mask the progression of a dual acute abdomen. Finally, anchoring the clinical picture with established severity scores, such as a high Alvarado score alongside TG18 Grade II cholecystitis, demonstrates that synchronous cases may not necessarily present with amplified systemic shock (Grade III), but rather with a confusing combination of localized peritoneal signs that mandate cross-sectional imaging [7].

The patient’s presentation with deranged liver function tests necessitated a careful differential diagnosis regarding hepatobiliary pathology. While AC can cause mild liver enzyme elevations, marked rises in bilirubin and alkaline phosphatase often raise suspicion for choledocholithiasis, cholangitis, or primary hepatic disease. However, the absence of common bile duct dilatation on dual-modality imaging, combined with the rapid normalization of total bilirubin and liver enzymes postoperatively, effectively ruled out persistent biliary obstruction. This trajectory strongly suggests that the transient hepatic derangements were due to either a spontaneously passed biliary micro-stone or, more likely, sepsis-associated inflammatory cholestasis driven by the perforated appendix and pelvic purulence. Recognizing that the primary source of sepsis was unequivocally confirmed on CT, extensive workups, including MRCP, viral hepatitis screening, and blood cultures, were appropriately bypassed to expedite definitive operative source control.

Intraoperatively, we attempted laparoscopic appendectomy but converted to an open approach due to a perforated, necrotic appendix with pelvic pus and dense adhesions involving the gallbladder. We elected to defer cholecystectomy and plan an interval procedure, as we decided to address the most emergent of the two intraoperatively. This staged approach, though less common than simultaneous laparoscopic removal of both organs, is well described. In the review, 27 of 40 patients underwent laparoscopic cholecystectomy with appendectomy, 8 underwent open procedures, and others had staged or percutaneous interventions [4,5]. Our intraoperative decision is therefore consistent with recognized practice in cases of severe local inflammation.

Postoperatively, our patient developed a surgical-site infection at the appendectomy wound, necessitating readmission. Among the 36 patients with available outcome data in the literature, 5 developed complications, including wound infection, sepsis, and gastrointestinal leakage [4].

Histopathology confirmed acute perforated appendicitis with serositis. Perforated variants are uncommon but documented: in the pooled appendiceal histology, one case was perforated gangrenous appendicitis and another was perforated appendiceal diverticulitis [4]. Gallbladder histology in our patient was reported as chronic cholecystitis [4,6].

Pathogenetically, several mechanisms have been proposed: bacterial translocation from a perforated appendix to the biliary system, systemic inflammatory responses triggering gallbladder ischemia, or purely coincidental simultaneous inflammation [2,5]. Our case, with prolonged symptoms and biochemical evidence of hepatobiliary involvement, may reflect parallel inflammatory processes rather than secondary spread.

A critical differential diagnosis in this clinical scenario is whether the gallbladder inflammation was a primary, synchronous AC or simply a secondary reactive serositis driven by the ruptured appendix. Purulent exudate from a perforated appendix can track cephalad along the right paracolic gutter into Morison’s pouch, bathing the gallbladder. In a patient with incidental, asymptomatic cholelithiasis, this external inflammation can cause gallbladder wall thickening and pericholecystic fluid, perfectly mimicking primary AC on imaging. While the widespread fat stranding noted on our patient's CT scan supports the presence of generalized peritonitis, several factors strongly suggest a true synchronous hepatobiliary pathology rather than an *innocent bystander* effect. First, the patient's early clinical presentation included deranged liver function tests (elevated bilirubin and ALP), which are characteristic of intrinsic hepatobiliary inflammation and less likely to result solely from localized appendiceal peritonitis. Second, and most definitively, the interval cholecystectomy performed three months after the complete resolution of the intra-abdominal sepsis revealed a persistently thickened gallbladder wall with histopathological confirmation of chronic cholecystitis. If the initial presentation had been purely a reactive serositis, the gallbladder would be expected to revert to a normal state following the index appendectomy and washout. Therefore, we conclude this represents a genuine synchronous pathology [8].

Taken together, our patient's profile - middle-aged male, dual localization of pain, leukocytosis, dual imaging confirmation, appendectomy with deferred cholecystectomy, and subsequent wound infection - adds to the literature by reinforcing the heterogeneity of synchronous presentations and by demonstrating the importance of flexible intraoperative decision-making.

## Conclusions

Synchronous AC and perforated appendicitis is an exceptionally rare clinical entity that poses a significant diagnostic challenge due to overlapping symptoms. This case of a 49-year-old male highlights that in patients presenting with evolving, atypical, or diffuse abdominal pain accompanied by marked leukocytosis, a high index of clinical suspicion for dual pathology is warranted. Early utilization of multimodal imaging, specifically the combination of abdominal ultrasound and computed tomography, is paramount for an accurate preoperative diagnosis. Furthermore, while simultaneous laparoscopic removal is often the standard of care, this case underscores the necessity of flexible, individualized operative planning. In complex cases where severe inflammation, dense adhesions, or perforation are encountered intraoperatively, a staged surgical approach - prioritizing the most emergent pathology via appendectomy - may be considered a pragmatic initial strategy rather than forcing a simultaneous resection. Deferring the cholecystectomy allows for patient stabilization and minimizes the risk of iatrogenic biliary injury. Definitive biliary treatment and pathological confirmation can then be safely accomplished via interval cholecystectomy once the acute inflammation has subsided. Finally, clinicians must remain vigilant for postoperative complications, such as surgical site infections, which can frequently arise following the management of complex, dual-inflammatory processes.
